# Monthly Summary

**Published:** 1892-06

**Authors:** 


### Monthly Summary.

MEDICINE IN VEGETABLES.—The following information may be useful to some, if not new to many: Spinach has a direct effect on the kidneys; a common dandelion used as greens is excellent for the same trouble; asparagus purges the blood; celery acts admirably on the nervous system, and is a cure for rheumatism and neuralgia; tomatoes act on the liver, beets and turnips are excellent appetizers; lettuce and cucumbers are cooling in their effects on the system, onions, garlic, leeks, olives and shallots, all of which are similar, possess medicinal virtue of a marked character, stimulating the circulatory system, and the consequent increase in the saliva and gastric juices, promoting digestion; red onions are an excellent diuretic, and the white ones are recommended to be eaten raw as a remedy for insomnia. A soup made of onions is regarded by the French as an excellent restorative in debility of the digestive organs.—*Herald of Health.*

---

THE WAY TO RAISE A TOOTHACHE.—"If any one really wishes to raise a toothache that shall cause him to be remembered, let him put arsenical paste in a wet cavity, the pulp being covered with refuse matter and decayed dentine, and then let him cap the climax of the outrage by thrusting into the outer cavity cotton wet with a sandarach solution. This will permeate the whole cavity, encapsule the arsenical paste, and prevent its action, while it serves as a constant irritant. In a few hours it will decompose, and the cavity will become foul almost beyond conception. We think it is really the worst covering for a temporary dressing of which we have any knowledge, and we have had experience enough with it to be expert in judging its demerits."*Dr. W. C. Barrett.*

---

MANIPULATING CEMENTS.—Dr. Wassall says: "I think there are two secrets in the use of cements that will give us better success with them. First, to take just enough of the plastic material on the instrument to fill the cavity, and burnish it until it is exactly full, so that it will not need to be cut or ground down after it has hardened. This leaves a surface which has an outside gloss to it, of a condensed vitreous nature. The other is to give the cement more time in setting before the rubber-dam is removed. It was Dr. Harlan who told that he allowed the rubber to stay on, as an invariable rule, one hour, after making a cement filling, and I have found it of great value.—*Ohio Journal.*

ACUTE CORYZA.—Dr. M. D. Lederman, of New York, finds the following mode of treatment very beneficial during the congestive stage of an acute nasal catarrh. The nasal chambers are sprayed with any of the antiseptic solutions, Seiler's preferred, until they are sufficiently cleansed, and then the following solution is used:

R/ Cocaine - - - -
Menthol, - - - aa gr. xx
Benzoinol - - - - ℥ ij
M. Ft. solution.

The feeling of fullness in the nose, associated with the dull frontal headache, is no doubt the result of extreme congestion. For the relief of these symptoms, cocaine has been used for a considerable period, but it has been found that its effect soon passes off, and the patient is left in the same uncomfortable position as before. To obviate this menthol was added to the solution. Menthol is a local anæsthetic, and in combination with cocaine, it keeps up the local depletion and leaves a pleasant coolness in the nasal chambers, enabling the patient to breath through the natural passages. Benzoinol is used in this solution as the menstruum, on account af its bland and unirritating qualities, and freedom from unpleasant odor.—*Brooklyn Medical Journal.*

---

THE CONTRACTION OF RUBBER-PLATES.—The fact is well established that vulcanite contracts in cooling, and, in consequence, dental plates made up with section teeth almost invariable warp, and require more or less manipulation before a satisfactory fit is secured. In the case of upper plates, the change is quite apparent, the rear palatal portion being thrown up, causing the plate to rock. The arching up of this part of the plate is caused by the contraction of that portion immediately behind the teeth, the thin palatal part acting as a stay, and diminishing to some extent the amount of change experienced.

When, in repairing an upper plate, the center portion is sawed out, it will be found that its heels will spring together, certainly as much as the amount removed by the saw cut, and sometimes even more. This shows that the same action takes place with lower plates, and to a greater extent than with upper ones. As they leave the vulcanizer, full lower plates, with section teeth, are always sprung together at the heels, and are too narrow for the mouth. If they are re-vulcanized, they are thereby made still narrower, and are. thereafter, in many cases, not capable of being worn with comfort. If they are heated sufficiently to soften the rubber and are then widened, the beneficial effect upon the fit will be quite apparent.—*Dr. Snow in Practitioner and Advertiser.*

---

CLEAR SHELLAC VARNISH.—To get an absolutely clear solution of shellac has long been a desideratum, not only with microscopists, but with all others who have occasional need of the medium for cements, etc. It may be prepared by first making an alcoholic solution of shellac in the usual way; a little benzole is then added, and the mixture well shaken. In the course of from twenty-four to forty-eight hours, the fluid will have separated into two distinct layers, an upper alcoholic stratum, perfectly clear, and of a dark-red color, while under it is a turbid mixture containing the impurities. The clear solution may be decanted or drawn off with a pipette.—*National Druggist.*

---

POISON OF THE TEETH.—Biting the nails is an exceedingly dangerous practice, as the biter never knows when to stop, and at any moment is liable to bite into "the quick" and cause blood poisoning. Even when the utmost care is taken of the teeth a poisonous secretion is apt to collect on them, and the entrance of a minute portion of this into the circulation may prove as certainly fatal as the pus on a surgeon's scalpel.—*Boston Herald.*

